# Systematic Identification of Cell-Wall Related Genes in *Populus* Based on Analysis of Functional Modules in Co-Expression Network

**DOI:** 10.1371/journal.pone.0095176

**Published:** 2014-04-15

**Authors:** Bin Cai, Cheng-Hui Li, Jian Huang

**Affiliations:** 1 Department of Genetics, Medical College of Soochow University, Suzhou, Jiangsu, People's Republic of China; 2 Department of Horticulture, Suzhou Polytechnic Institute of Agriculture, Suzhou, Jiangsu, People's Republic of China; Queen's University Belfast, United Kingdom

## Abstract

The identification of novel genes relevant to plant cell wall (PCW) biosynthesis in *Populus* is a highly important and challenging problem. We surveyed candidate *Populus* cell wall genes using a non-targeted approach. First, a genome-wide *Populus* gene co-expression network (PGCN) was constructed using microarray data available in the public domain. Module detection was then performed, followed by gene ontology (GO) enrichment analysis, to assign the functional category to these modules. Based on GO annotation, the modules involved in PCW biosynthesis were then selected and analyzed in detail to annotate the candidate PCW genes in these modules, including gene annotation, expression of genes in different tissues, and so on. We examined the overrepresented cis-regulatory elements (CREs) in the gene promoters to understand the possible transcriptionally co-regulated relationships among the genes within the functional modules of cell wall biosynthesis. PGCN contains 6,854 nodes (genes) with 324,238 edges. The topological properties of the network indicate scale-free and modular behavior. A total of 435 modules were identified; among which, 67 modules were identified by overrepresented GO terms. Six modules involved in cell wall biosynthesis were identified. Module 9 was mainly involved in cellular polysaccharide metabolic process in the primary cell wall, whereas Module 4 comprises genes involved in secondary cell wall biogenesis. In addition, we predicted and analyzed 10 putative CREs in the promoters of the genes in Module 4 and Module 9. The non-targeted approach of gene network analysis and the data presented here can help further identify and characterize cell wall related genes in *Populus*.

## Introduction

*Populus* represents the fast-growing temperate tree species in the world. The perennial growth habit and wide-ranging habitat of *Populus* makes it an important model woody crop for economic and ecological applications [1]. *Populus* supplies fiber resource for laminated veneer fabrication, and is widely used in the pulp and paper industry. Plant cell walls (PCWs) are mainly composed of polysaccharides and lignins, thereby becoming the basis of renewable resource. Thus, identifying which genes are involved in the formation and remodeling of PCWs is important for designing strategies to enhance desirable biomass properties.

Recently, the development of high throughput biological data collection techniques has led to the accumulation of a vast amount of ‘omics’ data, including complete genome sequences, transcript profiles, and so on. These data sets have been used to reconstruct networks and pathways to infer functional relationships among genes, proteins, and metabolites [2]. Many studies have constructed gene co-expression networks according to the similarity of gene expression patterns [3], [4], [5]. A gene co-expression network is an undirected graph that characterizes the gene neighborhood relationship at the systems level [6]. In a gene co-expression network, a node represents a gene, whereas an edge indicates the significant co-expression relationship between two genes. A gene co-expression network is constructed from the nonrandom dependencies of gene to gene expression calculated from various transcriptome perturbations. Co-expression network analysis has been applied to some plant biological problems, and has successfully generated biologically relevant hypotheses. Recently, several microarray analyses of *Populus* have been conducted using an Affymetrix Poplar GeneChip platform. These experiments were conducted under biotic, abiotic, and developmental conditions. The compendium of *Populus* microarray data has allowed the construction of a co-expression network.

Two strategies are usually used to inspect the functional relationships between genes. The first uses a guide-gene approach in which a set of genes with known functions, termed as guide genes (or bait genes), is used to retrieve related genes in the co-expression network based on the correlation coefficient analysis. Several previous studies have predicted PCW gene modules from gene co-expression networks in *Arabidopsis*, using a guide-gene approach [7], [8]. The other strategy uses a non-targeted approach (or top-down approach) to detect local modules from the entire network based on the topology of the links within the network. Genes involved in the same biological pathways or in the same functional complex tend to be clustered into a module; thus, module detection, followed by functional annotation, is one of the key steps in network analyses to infer gene functions. To date, reports on the determination of *Populus* cell wall genes using the non-targeted approach of gene network analysis are lacking.

Several transcription factors (TFs) belonging to NAC and MYB families have been proposed as key players in the regulation of plant secondary cell wall biosynthesis. These TFs interact with each other and form a complex regulatory network of secondary wall biosynthesis by interacting with cis-regulatory elements (CREs) in the target gene promoters [9]. Experimental elucidation of CREs related to PCW genes have been mainly carried out through forward genetic screening, which is time consuming and expensive [10]. The individual advancement in characterizing PCW-related CREs has been performed in *Arabidopsis*, but a more holistic view of these DNA motifs at the system level would be useful in developing strategies to further manipulate the PCW genes.

This study aims to survey the candidate *Populus* PCW genes in modules using a non-targeted approach. We collected 751 Affymetrix Poplar GeneChips available in the public domain to construct a genome-wide *Populus* gene co-expression network (PGCN). The co-expression modules were identified using a clustering method based on the connectivity of nodes in the network. To associate the modules with various biological processes, enrichment analysis of gene ontology (GO) terms was also performed to estimate the functional category for each particular co-expression module. The modules related to cell wall biosynthesis were selected based on the GO annotation. For the PCW-related modules, a detailed analysis was carried out to annotate the potential cell wall associated genes in the co-expression modules, including gene annotation, expression of genes in different tissues, and so on. Moreover, we examined the overrepresented motifs in the gene promoters to understand possible CREs in the promoters of co-expression genes within the functional module of cell wall biosynthesis. As deduced from neighbor genes in a module of cell wall formation, these overrepresented motifs carry regulatory information related to the TFs and co-expression genes involved in cell wall biology. These putative CREs provide candidates for further experimental identification of the functional CREs associated with PCW genes. This study would provide an efficient approach and a useful resource for discovering novel cell wall related genes in *Populus*.

## Materials and Methods

### Raw expression data

The *Populus* microarray data set was downloaded from GEO (http://www.ncbi.nlm.nih.gov/geo/), with the platform accession number of GPL4359. A total of 751 CEL files from GPL4359 were retrieved for the construction of the network. These CEL files were normalized with the RMA algorithm by the Affy library in the Bioconductor package [11]. Outlier detection by Boxplots using the arrayQualityMetrics tool [12] was performed to remove outlier samples. Arrays that failed the outlier tests were excluded from further analysis. Prior to network construction, control probe sets from the platform were removed.

### Probe sets and gene annotation

Probe sets from the Affymetrix Poplar GeneChip were mapped to the *P. trichocarpa* gene locus in Phytozome v9.1 (http://www.phytozome.net/poplar.php). The probes in a set were aligned to representative gene models using BlastN. A probe set was assigned to a gene if more than three probes from the set were perfectly aligned to the gene and the best hit (with the most number of matched probes) remained. When a probe set is mapped to multiple genes, the set was removed. When two or more probe sets matching a gene with same number of matched probes were present, one of the probe sets was randomly selected. The complete protein sequences of *Arabidopsis* and gene information were obtained from the *Arabidopsis* Information Resource (TAIR) release 10 (http://www.arabidopsis.org/). The *Populus* protein sequences were used to search the complete protein sequences of *Arabidopsis* using BlastP with an e-value cutoff of 1e-4 as [8], and the best hit, i.e., with the smallest e-value, was selected as *Arabidopsis* orthologs.

### Expression correlation calculation

The co-expression measure used in the network is similar to that applied in the calculation of gene co-expression data in ATTED-II (http://atted.jp/). Briefly, a correlation matrix of all gene expression was constructed by calculating pair-wise Pearson correlation coefficient (PCC) for genes using the normalized expression value across all samples. Redundancies and biases between samples were calculated as weight value for each sample. The weight value of each sample was used for calculating the PCC of a pair of gene expression profiles across all samples.

### Network analysis

Node degree (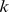) is the number of edges connecting to a node. Network density indicates the ratio of the observed number of edges to all possible edges in the network. The clustering coefficient (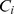) of a given node was calculated using the following formula: 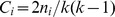, where 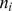 is the number of links connecting the 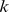 neighbors of node 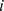 to each other. The function 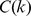 is the average clustering coefficient of all nodes with 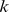 links. To identify functional modules in the network, we applied a clustering algorithm, Qcut [13], to partition the network.

### Functional enrichment

The GO annotation of the *Populus* genes was downloaded from agriGO (http://bioinfo.cau.edu.cn/agriGO/download.php). GO enrichment was performed within each module using BiNGO 2.4 [14]. The statistical significance of the GO term enrichment is measured by a hypergeometric test using the whole genome as the background/reference. A Bonferroni correction was used to regulate the false positive rate in the multiple testing problems.

### Analysis of gene expression

The *Populus* microarray data under different tissues were obtained from GEO, with the series number of GSE13990 (http://www.ncbi.nlm.nih.gov/geo/query/acc.cgi?acc=GSE13990). The tissues used include young leaves, mature leaves, roots, and xylem. The gene expression levels were expressed as 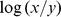, where 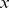 is the detection signal from the above tissue types and 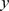 is the mean of tissue signals. Heat maps were generated using R (http://www.r-project.org/).

### Discovery of conserved motifs

To determine whether co-expressed genes in a module were transcriptionally co-regulated, we examined whether these genes share conserved CREs in their promoters. This test was carried out by developing a pipeline to identify the conserved sequence motifs in the promoter sequences of the relevant genes. First, we extracted an upstream region of 2000 bps from the translation start site as promoter sequences. Subsequently, the promoter sequences were submitted to the WeederTFBS [15]. The type of analysis is small, which allows motif lengths of 6 and 8 bps long. Each motif was allowed to appear more than once in a sequence. The enrichment of the highest scoring motifs from WeederTFBS was assessed by PWMEnrich package in R. The p-value was <0.05. Finally, to annotate the function of the overrepresented motifs, we compared these motifs with over 500 known plant cis-acting regulatory elements that have been curated in the PLACE database [16] using FASTA [17]. Motif degeneracy led to the 1 and 2 mismatches within the motifs of 6 and 8 bps over the whole length of sequence, respectively.

## Results and Discussion

### A pipeline for discovery of PCW genes

**Figure 1:**
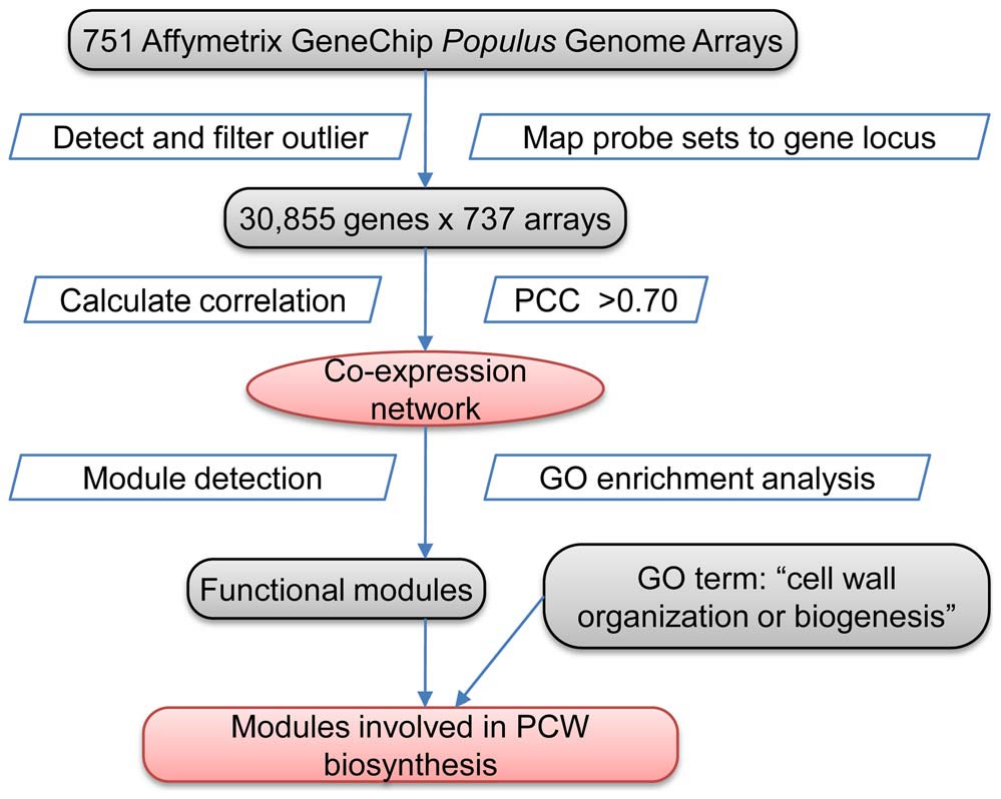
A flowchart of the computational analysis pipeline for discovery of plant cell wall genes.

To identify PCW genes using co-expression data sets, we developed a computational pipeline (Figure 1). The pipeline consists of the following steps: (1) construction of a genome-wide gene co-expression network; (2) identification of co-expression modules based on the connectivity of nodes in the network; (3) enrichment analysis of GO terms in functional modules; (4) selection and analysis of the modules related to cell wall biology.

### Application of no-targeted strategy to study PCW genes

In this study, we adopted a non-targeted approach to discover the genes in functional modules related to cell wall biogenesis directly in the *Populus* co-expression network. To assess the performance of no-targeted approach on discovery of PCW genes, we first predicted the modules responsible for cell wall biology in *Arabidopsis*. Since three secondary cell wall specific cellulose synthase genes, namely *AtCESA4*, *AtCESA7* and *AtCESA8*, were widely studied and well annotated [18], [19], we focus on the functional modules that contain these *CESA* genes. Using the pipeline developed in the study, we first constructed an *Arabidopsis* whole genome gene co-expression network (AGCN) with the *Arabidopsis* normalized gene expression values under 58 experiments from ATTED-II (http://atted.jp/download/GeneExp_v3.bz2). Using a PCC threshold of 0.65, the constructed co-expression network comprised 10,088 genes with 587,832 edges, and 381 modules were detected (Table S1).

In AGCN, Module 27 contains 52 genes and was significantly enriched with GO term “cell wall biogenesis” (FDR = 3.8e-47) and GO term “secondary cell wall biogenesis” (FDR = 1.8e-25) (Table S2). This module contains *AtCESA4*, *AtCESA7* and *AtCESA8* (Table S3).

These genes have been previously reported to be specifically involved in synthesis of cellulose in the secondary cell wall and are considered as specific markers for cells undergoing secondary cell wall formation [20], [21]. The module also contains many of the genes previously reported to be co-expressed with *CESA* genes, such as *GAUT12*, and *AtMYB46* [22], [23]. Therefore, Module 27 mainly responds to secondary cell wall biogenesis. In a recent study, using a guide-gene approach, 22 modules related to cell wall biogenesis were retrieved [8]. One of the modules, C02 (54 genes), was significantly enriched with “secondary cell wall biogenesis”.

We compared the Module 27 detected in AGCN with the C02 module to see if the no-targeted approach is workable in discovery of functional modules of PCW. There are 20 (38.5%) genes common to Module 27 and C02 (MCGs), 32 genes unique to Module 27 (MGs), and 38 genes unique to C02 (CGs). MCGs contains 7 bait genes used in [8], such as *AtCESA4*, *AtCESA7*, *AtCESA8*, *GAUT12*, *COBL4*, *FLA11*, and *AtMYB46*. It has been demonstrated that SND1 is a master transcriptional switch activating the developmental program of secondary wall biosynthesis and AtMYB103 is a direct target of SND1. *SND1* is common to Module 27 and C02, while *AtMYB103* is unique to Module 27. The MCGs are expressed preferentially in stem (i.e., the second internode counting from the bottom) (Figure S1A). To test whether the MGs and CGs show stem-specific expression patterns as MCGs, the expression patterns of MGs and CGs were also examined. All the 32 MGs are also expressed preferentially in stem, while some of the CGs is not mainly expressed in stem (Figure S1B; Figure S1C). The expression pattern of the MGs is more largely consistent with that of MCGs than CGs. Based on the expression pattern, Module 27 is likely to be more densely connected and all the genes show a stem-specific express pattern. This may be because the non-targeted approach facilitates the prediction of denser and more complete modules underlying significant biological process than the guide-gene approach. In the guide-gene approach, each sub-network included a seed gene and the genes with certain correlation with the seed gene. However, a lot of other connections with the neighbors of a seed gene cannot be captured to reduce noise and reveal underlying biological structure in most cases. The disadvantage of guide-gene approach is that the sub-network might be embedded within a larger module or it may include extra noisy genes [6]. Compared with the guide-gene approach, the non-targeted approach tends to detect the functional modules depending solely on the topological property of the network and can predict denser and more complete modules underlying significant biological process [13]. Although there is no hard evidence to tell whether CGs were co-expressed with MCGs, the assessment results on the Module 27 and C02 showed that no-targeted approach may be to predict denser functional modules, at least for the secondary cell wall biogenesis.

Furthermore, we compared all the predicted PCW genes with those predicted in two previous studies. Using 810 known/annotated PCW genes as guide genes, Wang et al. predicted 2,438 *Arabidopsis* cell wall genes [7]. Yang and his co-workers used 121 experiment validated *Arabidopsis* cell wall genes as guide genes and detected 694 PCW genes [8]. It is worth noting that the result of guide-gene approach depends on knowledge of biological processes, i.e. guide genes. In this study, a total of 31 modules responsive to cell wall biology were detected, consisting of 2,849 candidate PCW genes in *Arabidopsis*. In the 2,849 PCW genes, 851 (29.9%) genes are also previously reported and 1,998 genes were only predicted by this study (Figure S2). GO analysis was performed for the 1,998 new candidate PCW genes. Compared with the whole *Arabidopsis* genome annotation, biological processes related to cell wall biosynthesis were significantly enriched in these genes, including processes related to cell development, cell wall organization or biogenesis, polysaccharide biosynthesis (Table S4).

The 2,849 candidate PCW genes detected in this study were derived from the functional modules. Since these modules were detected from a robust whole genome-wide gene co-expression network, the 2,849 PCW genes may be better organized in various functional modules than those predicted by using a guide-gene approach. Therefore, the no-targeted approach used in this study is likely to predict some novel genes that omitted by guide-gene approach, and produce some denser and more complete functional modules.

### Co-expression network in *Populus*

PGCN was constructed from Affymetrix Poplar Genome Array samples obtained from the NCBI GEO repository. The experimental conditions under which the expression was measured included biotic and abiotic treatments, development, and so on (Table S5). After the removal of outlier samples, the PGCN was constructed using 737 microarray samples. These diverse sources of microarrays allow the determination of the true co-expression relationship between two genes. The microarray contains 61,313 probe sets after the control probe sets were removed. A total of 30,855 probe sets were mapped to *Populus* gene locus in Phytozome.

**Figure 2:**
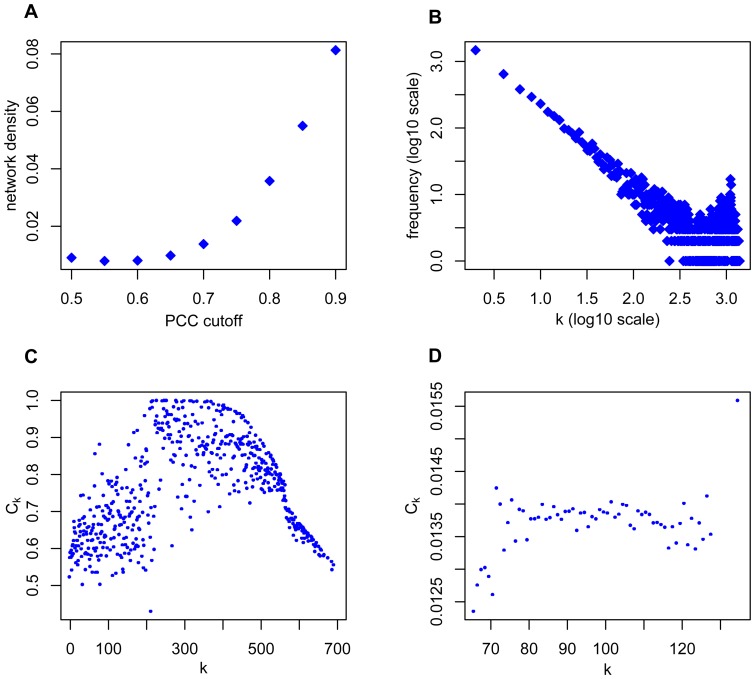
Properties of the PGCN. (A) Network densities at different PCC cutoff values. (B) Distribution of the node degree for PGCN. (C) The distribution of the clustering coefficient (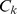) in relation to the node degree for PGCN. (D) The distribution of the 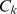 in relation to the node degree for random network.

Construction of the network started with the quantification of the expression profile similarities between gene pairs. To survey the possible redundancies and biases in the samples, the PCC between the samples was considered. Subsequently, the PCC between each pair of gene expression profiles was computed (see Materials and Methods). PCCs directly reflect the correlation of two genes, and setting the PCC cutoff is a key step to obtain significantly co-expressed genes. The changes in the network density at different PCC cutoff values were examined. As the cutoff value increases, the network density decreases in the low PCC range (<0.65), indicating that many nodes are connected by low-PCC links (Figure 2A). The network density then reaches a minimal value of approximately 0.65 PCC and increases thereafter. Above the PCC cutoff of 0.65, high PCC links will densely connect a decreasing number of nodes. As Aoki and his colleges suggested, biologically significant modules can be detected in the gene co-expressed network constructed with the PCC cut-off where the network density is greater than a minimal value [6]. A PCC cutoff value of 0.70 was chosen to significantly screen the co-expression correlation from a large-scale expression data set. The constructed network contains 6,854 genes with 324,238 edges.

The network follows the properties of biological networks, such as small world, scale free, and modular. The average node degree (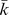) of PGCN is 95. The degree distribution of PGCN fits the power law (Figure 2B), and most nodes are connected only with one other node. The degree distribution of PGCN is scale free, similar to other co-expression networks in human, yeast, and *Arabidopsis*. The average clustering coefficient (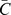) characterizes the overall tendency of the nodes to resemble a clique. In a real network, nodes appear as several modules of highly interconnected nodes. The density of connections between the neighbors of a node is shown by the clustering coefficient. The 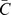 is an important measurement of the potential modularity of the network. The PGCN showed an 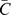 of 0.66. In a random network with the same number of nodes and links, 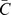 is 0.014. The distribution of 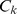 in relation to 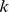 also distinguished the PGCN from the random network (Figure 2C; Figure 2D). For the PGCN, 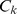 showed a complex distribution, whereas the 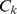 of the random network was approximately constant in relation to 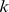. The 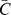 of PGCN is significantly larger than that of the random network, and the relationship between 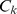 and 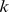 is more complex. These evidences indicate the potential modularity in the co-expression network. The small world, scale free and modular properties of the networks indicated the robustness of the PGCN.

We are mainly interested in modules enriched by GO terms of PCW formation and remodeling. The PCW gene modules and the other modules obtained in this study will also provide useful resources for the discovery of novel genes associated with certain biological problems in *Populus*.

### Detection and annotation of modules in PGCN

The constructed co-expression network remains complex and its biological functions are still difficult to understand. In the co-expression network, densely connected sub-networks are presented, and genes in the same module are co-expressed across diverse conditions, and thus, functional coherence among the genes in the module is expected. A module detected from the network is a more tightly interconnected structure, which would be more biologically meaningful and resistant to data noise. One of the most important analyses of co-expression network is to identify the sub-networks that represent functional modules of genes involved in a coherent biological process. The enrichment of GO biological processes for these modules revealed that the genes in a module have specific biological function. The partitioning analysis resulted in the identification of 435 modules, with the number per module ranging from 2 to 1335. The total number of nodes in all modules is 6,824. The smallest module contains only two nodes, and 234 (54.0%) of these modules were found. The genes in each module and the PCC values between genes are stored in Table S6.

Module detection followed by GO annotation is one of the key steps in network analyses to infer gene functions. Identification of overrepresented biological process GO terms can clarify the functional features of each module. In the whole-genome of *Populus*, 10,391 genes were identified by biological process GO terms. BiNGO 2.4 was adopted for GO term enrichment analysis of a selected gene set against a custom annotated gene set. We performed a biological process GO enrichment analysis in 192 modules containing at least 3 members using the whole genome genes with biological process GO terms as a custom reference set of GO annotations. Consequently, 67 modules were identified by the significantly overrepresented GO terms with false discovery rate-adjusted p-value (FDR)<0.05 of the hypergeometric test (Table S7).

### Co-expression modules involved in cell wall formation

Understanding the molecular basis of cell wall formation is vital for designing strategies for enhancing desirable biomass properties. We focused on modules involved in cell wall formation. The GO term “cell wall organization” or “cell wall biogenesis” was assigned to 6 modules.

#### Module 4

Module 4 contains 354 genes with 5,459 edges and was also significantly enriched with GO term “secondary cell wall biogenesis” (FDR = 9.9e-11). The functional annotation of all genes was based on the similarity searching of *Arabidopsis* genes in TAIR10. A total of 340 (96.1%) genes have homologs in *Arabidopsis*; among which 168 (49.4%) have *Arabidopsis* homologous genes with known function (Table S8). This module includes 5 genes encoding CESA and 2 genes encoding cellulose synthase-like (CSL). Two kinds of TFs (NAC and MBY) are involved in this module (Table 1). 6 genes encode homologous genes of *SND1*, *SND2*, *NST1* and *VND1*. Module 4 includes 15 genes having significant homology with *AtMYB42*, *AtMYB46*, *AtMYB50*, *AtMYB52*, *AtMYB69*, *AtMYB83*, *AtMYB86* and *AtMYB103*. In Module 4, the expression of genes is mainly in the xylem (Figure S3A).

**Table 1:** *Populus* transcription factors in Module 4.

		*Arabidopsis* gene		
*Populus* gene	Probe set ID	Locus	Name	E-value
Potri.011G153300.1	PtpAffx.50281.1.S1_at	AT1G32770	ANAC012/NST3	4e-116
Potri.005G116800.1	PtpAffx.161809.1.S1_at	AT2G18060	ANAC037/VND1	8e-168
Potri.002G178700.1	PtpAffx.202301.1.S1_at	AT2G46770	ANAC043/NST1	6e-136
Potri.001G448400.1	Ptp.1762.1.A1_at	AT2G46770	ANAC043/NST1	2e-115
Potri.007G135300.1	PtpAffx.206916.1.S1_at	AT4G28500	ANAC073/SND2	9e-129
Potri.017G016700.1	PtpAffx.15627.2.S1_s_at	AT4G28500	ANAC073/SND2	3e-133
Potri.003G132000.1	PtpAffx.224312.1.S1_x_at	AT1G63910	AtMYB103	1e-107
Potri.001G099800.1	PtpAffx.224153.1.S1_s_at	AT1G63910	AtMYB103	3e-108
Potri.015G129100.1	PtpAffx.162910.1.S1_at	AT4G12350	AtMYB42	4e-88
Potri.003G114100.1	Ptp.6319.1.S1_at	AT4G12350	AtMYB42	4e-105
Potri.012G127700.1	PtpAffx.2427.1.S1_at	AT4G12350	AtMYB42	2e-89
Potri.001G258700.1	PtpAffx.200828.1.S1_x_at	AT5G12870	AtMYB46	5e-73
Potri.009G053900.1	PtpAffx.204871.1.S1_x_at	AT5G12870	AtMYB46	5e-75
Potri.015G082700.1	PtpAffx.147746.1.A1_at	AT1G57560	AtMYB50	6e-83
Potri.005G186400.1	PtpAffx.205579.1.S1_at	AT1G17950	AtMYB52	2e-66
Potri.002G073500.1	PtpAffx.201825.1.S1_at	AT1G17950	AtMYB52	6e-66
Potri.007G134500.1	PtpAffx.206921.1.S1_at	AT1G17950	AtMYB52	4e-82
Potri.015G033600.1	Ptp.116.1.S1_s_at	AT1G17950	AtMYB52	1e-85
Potri.007G106100.1	PtpAffx.207054.1.S1_at	AT4G33450	AtMYB69	2e-70
Potri.001G267300.1	PtpAffx.224175.1.S1_s_at	AT3G08500	AtMYB83	1e-91
Potri.012G084100.1	PtpAffx.224807.1.S1_s_at	AT5G26660	ATMYB86	3e-82

#### Module 9

Module 9 contains 122 genes with 739 edges, and was significantly enriched with GO term “cellular glucan metabolic process” (FDR = 3.3e-03). In this module, 119 (97.5%) genes have homologous genes in *Arabidopsis*; among which, only 59 (49.6%) can be functionally annotated; and the remaining were classified as uncharacterized protein (Table S9). The expressions of genes in Module 9 display diverse pattern in various tissues (Figure S3B).

#### Other modules related to cell wall biogenesis

Module 12 consists of 62 genes, with the most significant GO term “cell wall organization” (FDR = 1.0e-02). Module 40 contains 20 genes, with the most significant GO term “sucrose metabolic process” (FDR = 2.7e-02). This module includes a homologous gene of *AtCESA3*. Module 52, consisting of 16 genes, was involved in cell growth process (FDR = 3.8e-02). Module 63 (14 genes) was associated with defense response to fungus process (FDR = 3.1e-033).

#### Module 4 and 9 represent two different processes in cell wall biology

Both the genes in Module 4 and Module 9 are important for biological processes contributing to the cell wall formation. The GO annotation of the two modules preliminary revealed that Module 4 is composed of genes in the secondary cell wall biogenesis, whereas Module 9 is mainly involved in cellular polysaccharide metabolic processes in the primary cell wall. Cellulose, a key structural component of the plant cell wall, is synthesized at the plasma membrane by membrane-localized “rosette” complexes [24]. Cellulose synthase (CESA) has been localized in these cellulose-synthesizing complexes. In *Arabidopsis*, *AtCESA4*, *AtCESA7* and *AtCESA8* are required for the development of the thick secondary wall [18], [19], [25]. It is reported that *AtCESA4*, *AtCESA7* and *AtCESA8* genes are specifically involved in synthesis of cellulose in the secondary cell wall and are considered as specific markers for cells undergoing secondary cell wall formation [20], [21]. Module 4 includes four genes encoding CESA4, CESA7 and CESA8. Besides, the *NAC* and *MYB* genes in Module 4 have been experimentally verified as master transcriptional switches that activate the developmental program of secondary wall biosynthesis [9]. In many dicotyledonous plants, the polysaccharide xyloglucan (XG) is the most abundant hemicellulose in the primary cell wall. The xyloglucan endotransglucosylase/hydrolase (XTH) family of proteins is believed to cut and ligate XGs, as a means of integrating new XGs into the cell wall [26]. Module 9 includes four members of genes putatively encoding XTH proteins. The expression patterns of genes in Module 9 are different from that of the members in Module 4, as shown on the heat map of the expression patterns from microarray data of young leaves, mature leaves, roots, and xylem. The genes in Module 4 are preferentially expressed in the xylem, whereas Module 9 displays diverse pattern. Therefore, Module 4 is involved in cellular polysaccharide metabolic process in the secondary cell wall, whereas Module 9 comprises genes involved in primary cell wall biogenesis. Wood or the secondary xylem in trees produces most of the biomass. This lignocellulosic pool offers an enormous and renewable polysaccharide feedstock for materials and biofuels. The genes in Module 4 are the main targets for biomass manipulation.

### Overrepresented putative CREs

To verify whether co-expressed genes in Module 4 and Module 9 are transcriptionally co-regulated, we examined whether these genes shared conserved sequence motifs as potential regulatory elements in their promoters, using a pipeline described in the Materials and Methods. For each dataset, we detected motifs by WeederTFBS, a tool for discovering conserved motifs in a set of related DNA sequences. Only the overrepresented motifs are possibly related to the function of the TF, and thus, we performed the enrichment assessment of the motifs using PWMEnrich package. The overrepresented motifs are considered putative CREs in modules. Overall, we found five CREs in both Module 4 and Module 9, respectively (Table 2), providing a strong evidence that most genes in the same module are transcriptionally co-regulated.

**Table 2:** Overrepresented cis-regulatory elements in Module 4 and Module 9 of PGCN.

Module	Motif	P value	Consensus sequence	PLACE accession	PLACE cis-element function
4	1	6.8e-5	ACCCCC	S000193	ACII element; Myb protein binding sites; Vascular-specific expression
4	2	9.6e-5	GAGGGG	S000288	SE1 (Stem element 1);Vascular expression
4	3	1.1e-3	CAGGGGGG		
4	4	2.0e-3	CACCCC	S000306	elicitor and light responsive cis-acting elements
4	5	1.5e-2	AGGGGGGC	S000437	Binding site of R2R3-type MYB factor
9	1	6.8e-8	CCCCTCCT	S000221	CE3 (coupling element 3);ABA responsive element
9	2	7.8e-8	CCCTCC	S000192	ACII element; Myb protein binding sites;Vascular-specific expression
9	3	2.8e-6	CCCCTC	S000189	required for phloem-specific gene expression
9	4	6.9e-3	GAGGGGGG		
9	5	4.5e-2	CCCCCCCT	S000192	ACII element; Myb protein binding sites;Vascular-specific expression

To annotate these predicted CREs, we compared them with known CREs in the PLACE database. A total of 7 CREs matched with known cis-regulatory motifs. In Module 4, three CREs were annotated as SE1 (S000288), ACII element (S000193) and S000437, respectively, which are vascular-specific elements. In Module 9, CREs showed similarity to the elements involved in hormone response (S000221), vascular specificity (S000192), and required for phloem-specific gene expression (S000189). Motif 1 and Motif 5 in Module 4 and Motif 5 in Module 9 were annotated as the known MYB protein binding sites in the PLACE database, which are required for vascular-specific gene expression.

### TFs and CREs in Module 4

**Figure 3:**
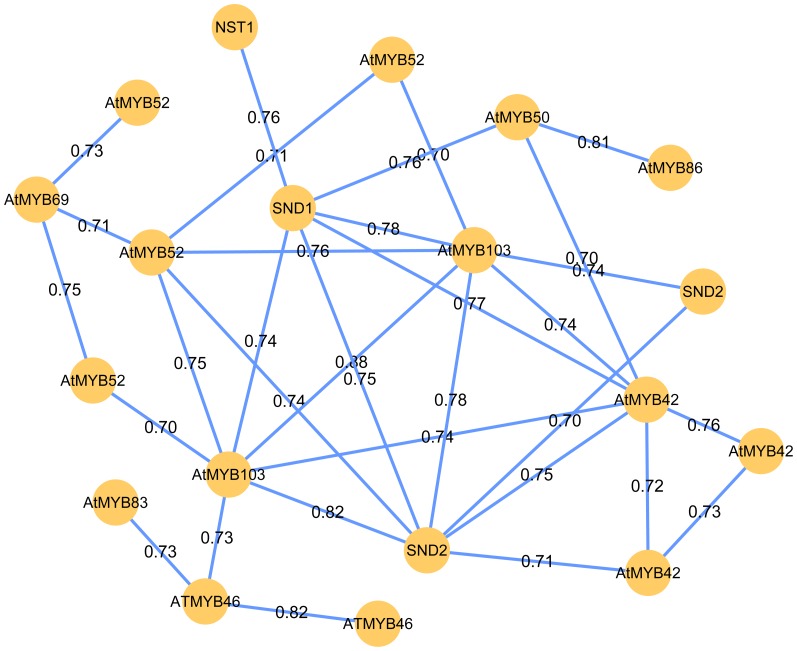
Co-expression relationship between transcription factors (TFs) in Module 4 of PGCN. A node represents a TF gene and an edge indicates a significant co-expression relationship between two genes. The gene name of the *Arabidopsis* homologous gene is used for each TF.

In *Arabidopsis*, evidences indicated that SND1 is a master switch that activates the developmental program of secondary wall biosynthesis. In Module 4, the homologs of *NST1* (Potri.001G448400.1), *SND2* (Potri.007G135300.1), *AtMYB42* (Potri.003G114100.1) and *AtMYB103* (Potri.001G099800.1 and Potri.003G132000.1) were directly connected to *SND1* (Potri.011G153300.1) (Figure 3). This result agrees with that SND1 and NST1 redundantly regulate the secondary wall thickening in fibers, and SND2, MYB46 and MYB103 was identified as a direct targets of SND1 in *Arabidopsis* [9], [27]. The members of *NAC* and *MYB* gene families in Module 4 are co-expressed across diverse conditions, suggesting the potential underlying co-regulation mechanism. The connectivity of these genes in this gene co-expression network will allow the investigation of the interaction between *NAC* and *MYB* genes through further approaches of experiment.

Typically, gene expression is mediated by TFs, which bind to specific DNA sequences (transcription factor binding sites, TFBSs) [28]. PGCN provides the co-expressed relationship between TFs and other members in the module. The genes in Module 4 are involved in regulating secondary wall biosynthesis and become the most important targets with respect to production of biomass. Understanding regulatory pathways in Module 4 is important for selecting and manipulating genes associated with the production of biomass. It is necessary to characterize regulatory elements in the promoter sequences of co-expressed genes in the module. Notably, 5 putative CREs are overrepresented in the specific dataset relative to a background. These motifs are functional CREs necessary for or related to coordinated regulation of these co-expression genes, especially the TFs, because these co-expression genes are adjacent to the TFs. In Module 4, 15 *MYB* genes are found, and 2 putative CREs matched well with known MYB protein binding sites required for vascular-specific gene expression. This result indicated that these putative CREs are likely to be the TFBSs of the MYB TFs. These motifs are involved in secondary cell wall biology, providing candidates for the further experimental verification of TFBSs and other related functional CREs associated with the TFs.

### Comparison with previous studies

In a previous study, 817 candidate *Populus* cell wall genes were obtained by querying of the *Populus* genome using the 692 *Arabidopsis* cell wall genes, which were predicted by using a guide-gene approach based on co-expression network [8]. In this study, we first constructed a genome-scale gene co-expression network and partitioned the network into the modules based on the topological property of the network in *Populus*. Subsequently, based on the functional annotation of each module using GO enrichment analysis, we selected 6 modules related to cell wall biology, covering 588 genes. Given that genes in the same module are densely connected and co-expressed across diverse conditions, functional coherence among the genes in the module is expected. Thus, we presume that the 588 *Populus* genes are candidate PCW genes, 81% of which are included in two modules, Module 4 and Module 9. In these PCW genes, only 96 (16.3%) genes were also reported by the previous study and 492 genes were new candidates predicted by this study. GO enrichment analysis was performed for the 492 new candidate PCW genes and biological processes related to cell wall biosynthesis were significantly enriched in these genes, including processes related to cellular glucan metabolic process, polysaccharide metabolic process, plant-type cell wall biogenesis, plant-type cell wall organization, and so on (Table S10). Compared with the PCW genes predicted by homology search, the PCW genes found in this study were directly derived from a robust whole genome gene co-expression network in *Populus* and clustered as complete and dense functional modules.

## Supporting Information

Figure S1 **Tissue-specific expression patterns (based on microarray data) of (A) 20 genes common to both Module 27 in AGCN and C02 in** [8] **(MCGs), (B) 32 genes unique to Module 27 (MGs), and (C) 38 genes unique to C02 (CGs).**
(PDF)

Figure S2 **Number of unique and common candidate** ***Arabidopsis*** **cell wall genes found in this study and previous studies.** A: AGCN, B: [8], C: [7].
(PDF)

Figure S3 **Heat map visualization of gene expression patterns of members in (A) Module 4, and (B) Module 9 in different tissues of** ***Populus*** **.** The expression data was derived from a microarray deposited in GEO, and the series number is GSE13990. Color scale at the top of the dendrogram represents log2 ratio value of treated sample to control sample.
(PDF)

Table S1 **List of the significant co-expression relationships among genes in each module in AGCN.**
(XLSX)

Table S2 **Significantly overrepresented biological process GO terms in AGCN modules.**
(XLSX)

Table S3 ***Arabidopsis*** **genes of Module 27 in AGCN.**
(XLSX)

Table S4 **GO enrichment for the 1,998 new candidate plant cell wall genes found in AGCN.**
(XLSX)

Table S5 **Experiment series of** ***Populus*** **gene chips used for co-expression network construction.**
(XLSX)

Table S6 **List of the significant co-expression relationships among genes in each module in PGCN.**
(XLSX)

Table S7 **Significantly overrepresented biological process GO terms in PGCN modules.**
(XLSX)

Table S8 **Best-hit homologous genes in** ***Arabidopsis*** **of the** ***Populus*** **genes in Module 4.**
(XLSX)

Table S9 **Best-hit homologous genes in** ***Arabidopsis*** **of the** ***Populus*** **genes in Module 9.**
(XLSX)

Table S10 **GO enrichment for the 492 new candidate plant cell wall genes found in PGCN.**
(XLSX)
