# Supplementary figures and images for: Systematic Identification of Cell-Wall Related Genes in Populus Based on Analysis of Functional Modules in Co-Expression Network
Source: PLoS One. 2014 Apr 15;9(4):e95176. doi: 10.1371/journal.pone.0095176 (PMC3988181; doi:10.1371/journal.pone.0095176)

A

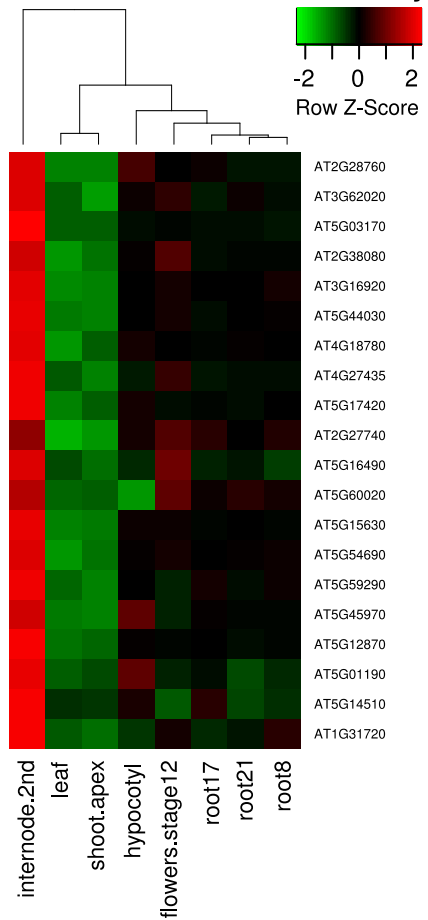

B

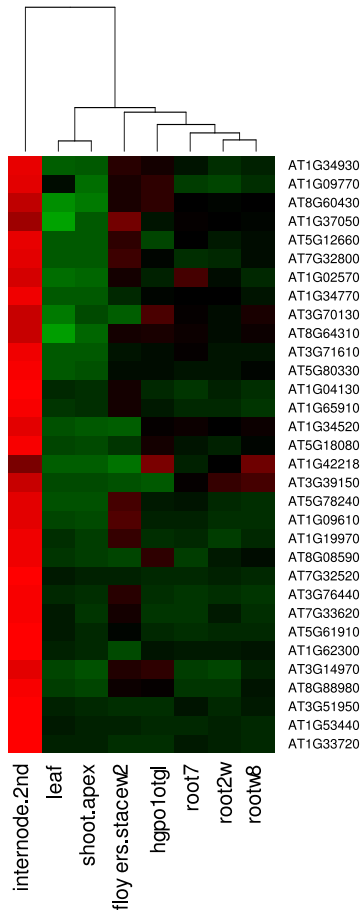

C

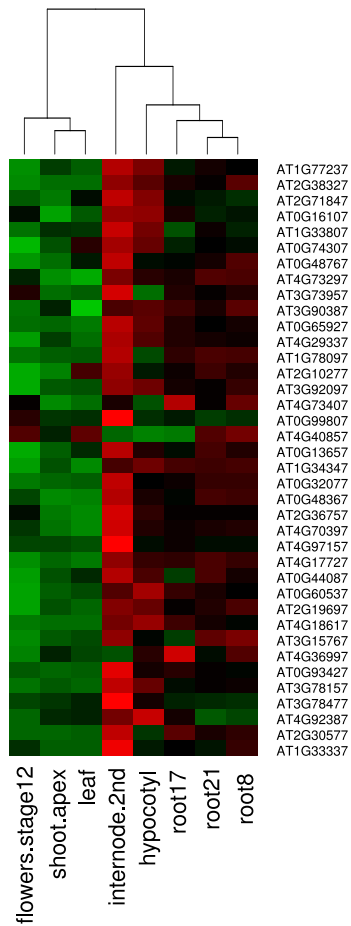

Supplement: Figure S1 — Tissue-specific expression patterns (based on microarray data) of (A) 20 genes common to both Module 27 in AGCN and C02 in [8] (MCGs), (B) 32 genes unique to Module 27 (MGs), and (C) 38 genes unique to C02 (CGs). (PDF) [file pone.0095176.s001.pdf]

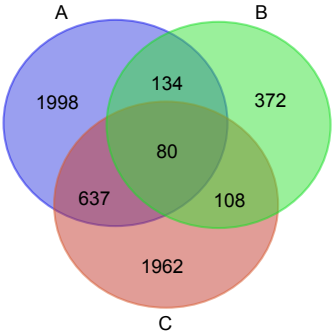

Supplement: Figure S2 — Number of unique and common candidate Arabidopsis cell wall genes found in this study and previous studies. A: AGCN, B: [8], C: [7]. (PDF) [file pone.0095176.s002.pdf]
