# Supplementary material for: Systematic Identification of Cell-Wall Related Genes in Populus Based on Analysis of Functional Modules in Co-Expression Network
Source: PLoS One. 2014 Apr 15;9(4):e95176. doi: 10.1371/journal.pone.0095176 (PMC3988181; doi:10.1371/journal.pone.0095176)
Supplement: Figure S3 — Heat map visualization of gene expression patterns of members in (A) Module 4, and (B) Module 9 in different tissues of Populus . The expression data was derived from a microarray deposited in GEO, and the series number is GSE13990. Color scale at the top of the dendrogram represents log2 ratio value of treated sample to control sample. (PDF) [file pone.0095176.s003.pdf]

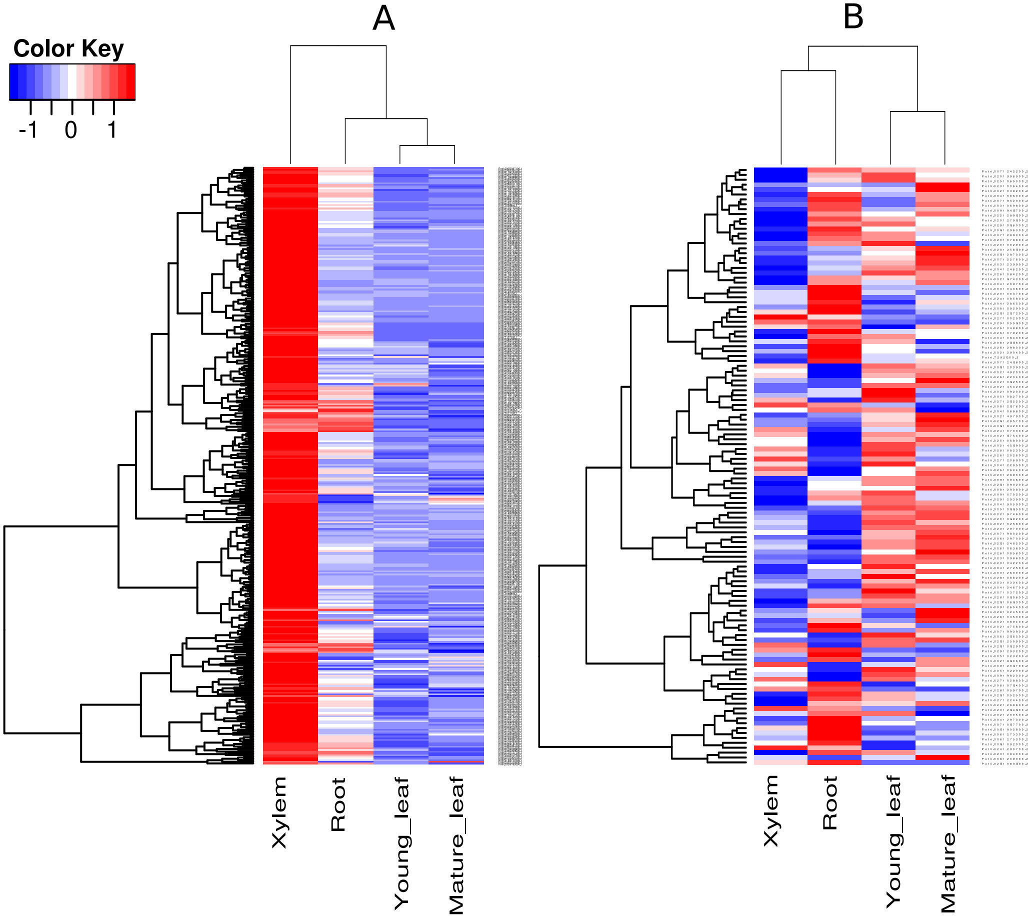
A
B
Color Key
-1
0
1
Xylem
Root
Young_leaf
Mature_leaf
Xylem
Root
Young_leaf
Mature_leaf
